# Effect of Time-Restricted Eating on Sleep in Type 2 Diabetes

**DOI:** 10.3390/nu16162742

**Published:** 2024-08-17

**Authors:** Vasiliki Pavlou, Shuhao Lin, Sofia Cienfuegos, Mark Ezpeleta, Mary-Claire Runchey, Sarah Corapi, Kelsey Gabel, Faiza Kalam, Shaina J. Alexandria, Alaina P. Vidmar, Krista A. Varady

**Affiliations:** 1 Department of Kinesiology and Nutrition, University of Illinois Chicago, Chicago, IL 60612, USA; pavlou2@uic.edu (V.P.); slin89@uic.edu (S.L.); scienf2@uic.edu (S.C.); mrunch@uic.edu (M.-C.R.); scorap2@uic.edu (S.C.); kdipma2@uic.edu (K.G.); 2 Division of Endocrinology, Metabolism and Diabetes, University of Colorado School of Medicine, Aurora, CO 80045, USA; mezpel2@uic.edu; 3 Division of Cancer Prevention and Control, Department of Internal Medicine, College of Medicine, The Ohio State University, Columbus, OH 43201, USA; 4 Department of Preventative Medicine (Biostatistics), Northwestern University, Chicago, IL 60208, USA; shaina.alexandria@northwestern.edu; 5 Center for Endocrinology, Diabetes and Metabolism, Department of Pediatrics, Children’s Hospital Los Angeles, Keck School of Medicine of the University of Southern California, Los Angeles, CA 90033, USA; avidmar@chla.usc.edu

**Keywords:** time restricted eating, intermittent fasting, type 2 diabetes, sleep quality, sleep duration, insomnia, obstructive sleep apnea

## Abstract

The aim of this secondary analysis was to compare the effects of time-restricted eating (TRE) versus calorie restriction (CR) and controls on sleep in adults with type 2 diabetes (T2D). Adults with T2D (n = 75) were randomized to 1 of 3 interventions for 6 months: 8 h TRE (eating only between 12 and 8 pm daily); CR (25% energy restriction daily); or control. Our results show that TRE has no effect on sleep quality, duration, insomnia severity, or risk of obstructive sleep apnea, relative to CR and controls, in patients with T2D over 6 months.

## 1. Introduction

Short sleep duration (<5 h) and poor sleep quality are associated with the risk of developing type 2 diabetes (T2D), and this relationship is thought to be bi-directional [1]. Individuals with T2D have a higher occurrence of obstructive sleep apnea and can experience disturbances in sleep from diabetes-related nighttime symptoms, such as hypoglycemia, nocturia, or neuropathy [2,3]. Conversely, laboratory studies show a reduction in insulin sensitivity when sleep duration and quality are decreased in adults with T2D [4,5]. 

Weight loss may improve sleep in those with obesity and T2D. For instance, the Sleep AHEAD trial showed that weight loss of 10.4% in those with T2D improved obstructive sleep apnea symptoms and even led to sleep apnea remission for some participants after a 1-year intervention [6]. In terms of sleep quality and duration, a 2-year weight loss trial in adults with obesity (with and without diabetes) showed improvements in participants who lost more than 5% of their body weight at 6 months compared with those who lost less than 5% [7].

Time-restricted eating (TRE) is a weight loss intervention that involves confining food intake to 4 to 10 h per day and fasting for the remaining hours with energy-free beverages [8]. TRE results in an unintentional reduction in energy intake of ~300–500 kcal/d and weight loss of ~3–5% over 2–12 months in adults with obesity [8]. Smaller eating windows tend to produce a greater reduction in energy intake, leading to more significant weight loss. Therefore, TRE can serve as an alternative to calorie counting for weight management. 

Recently, our group showed that TRE is also effective for weight loss in patients with T2D [9]. What remains unknown, however, is whether these changes in body weight have any impact on sleep parameters in this population. Accordingly, we conducted this secondary analysis to compare the effects of 6 months of TRE versus CR and controls on sleep quality, duration, insomnia severity, and the risk of obstructive sleep apnea in adults with T2D. We hypothesized that the TRE group would achieve greater improvements in sleep quality and duration compared with the CR and control groups due to greater weight loss in TRE participants.

## 2. Methods

### 2.1. Trial Participants

This is a secondary analysis of a 6-month randomized controlled trial [9]. Participants were recruited from the Chicagoland area by flyers posted around the University of Illinois Chicago campus. Inclusion criteria: 18 to 80 years old; previous diagnosis of T2D; glycated hemoglobin (HbA1c) between 6.5–11.0%; and BMI between 30 and 50 kg/m^2^. Exclusion criteria: >4% weight loss or gain in the 3 months prior to the study start; eating within less than a 10 h eating window; pregnant or attempting to conceive; history of eating disorders; nightshift work; and current smoking. Written informed consent was provided by all participants and the protocol received approval from the Office for the Protection of Research Subjects at the University of Illinois Chicago (Protocol #2021-1086).

### 2.2. Intervention Groups

A stratified random sampling procedure based on BMI, age, sex, and HbA1c was used to randomize participants into 1 of 3 groups: TRE, CR, or control. TRE participants were instructed to eat ad libitum between 12:00 and 8:00 pm daily and fast from 8:00 to 12:00 pm the following day. Participants were not restricted on the quantities or types of food consumed within the 8 h eating window. TRE participants were allowed to consume energy-free beverages during the 16 h fasting period. CR participants were instructed to consume 75% of their baseline energy needs every day. The Mifflin equation [10] was used to calculate total energy expenditure at baseline. CR participants were asked to track their food intake daily using the MyFitnessPal app. Participants met with a study dietitian weekly to receive support in tracking accurately and adhering to their prescribed calorie goals. Control participants were instructed to remain weight stable by keeping their eating and activity routines unchanged.

### 2.3. Outcome Measures

All variables were assessed at baseline and month 6 [9]. Briefly, body weight was measured at the research center using a digital scale. Lean mass, fat mass, and visceral fat mass were measured by dual-energy X-ray absorptiometry (iDXA, GE). HbA1c levels were analyzed by a commercial lab (MedStar, Sparta, IL, USA). Time in euglycemic range (i.e., glucose levels between 70–180 mg/dL) was assessed using a continuous glucose monitor (CGM; Dexcom G6 Pro, San Diego, CA, USA). A pedometer (Fitbit, San Francisco, CA, USA) was used to assess physical activity (steps/d). Dietary intake (including caffeine intake) was measured using a 7 d food record by the NIH Automated Self-Administered 24 h Dietary Assessment Tool (ASA-24) [11].

Sleep quality, duration, and timing were measured using the Pittsburgh Sleep Quality Index (PSQI) [12]. The PSQI is a 19-item self-report questionnaire that measures sleep quality in the past month, resulting in a total score of 0–21. Poor sleep quality is indicated by scores above 5. PSQI was also used to measure sleep onset latency. The sleep latency score ranges from 0 to 3, with 0 indicating no problem falling asleep and 3 indicating a severe problem. Insomnia severity was assessed by the Insomnia Severity Index (ISI) [13]. The ISI is a 7-item self-report questionnaire that rates each item using a 5-point Likert scale, resulting in a total score of 0–28. Scores are stratified into the following categories: no clinically significant insomnia (0–7); subclinical insomnia (8–14); moderate severity insomnia (15–21); and severe insomnia (22–28). High risk of obstructive sleep apnea (% occurrences) was estimated using the 10-item self-report, i.e., the Berlin Questionnaire [14]. Chronotype was quantified by the Morningness–Eveningness Questionnaire (MEQ), for which scores are stratified as follows: definitely morning type (70–86), moderately morning type (59–69), intermediate type (42–58), moderately evening type (31–41), and definitely evening type (16–30) [15].

### 2.4. Statistical Analysis

Data are shown as mean (95% confidence interval [CI]) unless otherwise noted. We conducted an intention-to-treat analysis, which included data from all 75 participants who underwent randomization. A linear mixed model was used to assess time, group, and time*group effects for each outcome. In each model, time and group effects (and their interaction) were estimated without imposing a linear time trend. A Bonferroni-adjusted two-tailed *p*-value of less than 0.017 was considered statistically significant for pairwise group comparisons of percent change in body weight (primary outcome). *p*-Values generated from analyses of secondary outcomes were not adjusted for multiplicity and are considered descriptive. Pearson correlations were performed to assess the relationships between changes in body weight, HbA1c, and sleep measures. All analyses were performed using R software (v4.3.1).

## 3. Results

### 3.1. Participant Baseline Characteristics and Dropouts

As previously reported [9], a total of 127 participants were screened, and 75 were randomized to the TRE group (n = 25), CR group (n = 25), or control group (n = 25). By the end of the 6-month study, the number of completers was n = 69 (TRE: n = 23; CR: n = 22; control: n = 24). The baseline characteristics of the participants were comparable between groups (Table 1). Participants in the TRE, CR, and control groups were classified in the intermediate chronotype (i.e., MEQ score between 42 and 58).

### 3.2. Energy Intake

The mean (SD) energy deficit was −313 (SD 509) kcal/d in the TRE group, −197 (SD 426) kcal/d in the CR group, and −16 (439) kcal/d in the control group over 6 months. At this level of caloric restriction, we would expect the TRE group to have lost 6.8 kg by month 6 and the CR group to have lost 4.3 kg by month 6. These projections are based on the assumption that 1 kg of weight loss is roughly equivalent to a 7700 reduction in caloric intake. In actuality, our TRE participants lost 4.28 kg and our CR participants lost 2.50 kg by month 6 relative to baseline (Table 2). Since the projected weight loss was higher than the actual weight loss achieved, it can be postulated that our participants were underreporting food intake, which may have artificially inflated the degree of caloric restriction reported.

### 3.3. Body Weight and Body Composition

**Figure 1:**
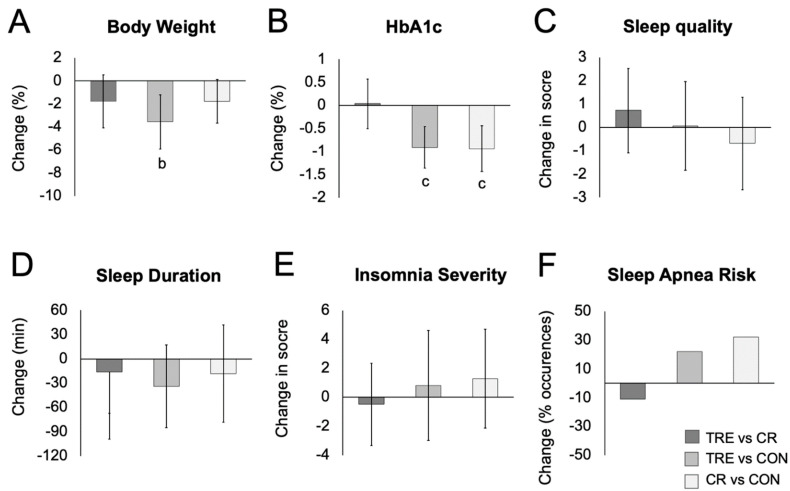
Change in body weight, HbA1c, and sleep parameters between TRE, CR, and control groups ^a^. Abbreviations: CON: control group, CR: calorie restriction group, HbA1c: glycated hemoglobin, TRE: time-restricted eating group. (**A**) Change in body weight between groups. (**B**) Change in HbA1c between groups. (**C**) Change in sleep quality score between groups. (**D**) Change in sleep duration between groups. (**E**) Change in insomnia severity score between groups. (**F)** Change in risk of obstructive sleep apnea between groups. ^a^ Means were estimated using an intention-to-treat analysis using a linear mixed model with 95% CIs for each parameter from baseline by group. ^b^ Indicates statistical significance using Bonferroni-adjusted two-tailed *p* < 0.017. ^c^ Indicates statistical significance using *p* < 0.05.

Changes in body weight and body composition are displayed in Table 2 and Figure 1. By month 6, body weight significantly decreased in the TRE group (−3.56%; 95% CI, −5.92 to −1.20%), but not in the CR group (−1.78%, 95% CI; −3.67 to 0.11%) versus controls. Fat mass decreased in the TRE group by month 6 (−2.49 kg; 95% CI, −4.41 to −0.58 kg) but not in the CR group (−1.65 kg; 95% CI, −3.33 to 0.04 kg) relative to controls. Waist circumference was reduced in the TRE group (−3.44 cm; 95% CI, −5.71 to −1.18 cm) and CR group (−3.50 cm; 95% CI, −5.80 to −1.20 cm) relative to controls, with no differences between TRE and CR. Visceral fat mass and lean mass did not change significantly between groups.

### 3.4. Glucoregulation

Changes in glucoregulation are displayed in Table 2 and Figure 1. HbA1c levels significantly decreased in the TRE group (−0.91%; 95% CI, −1.61 to −0.20%) and CR group (−0.94%; 95% CI, −1.59 to −0.30%) relative to controls, with no differences between the TRE and CR groups. Time in euglycemic range increased in the CR group by month 6 (18.46%; 95% CI, 0.31 to 36.62%) but not in the TRE group (12.59%; 95% CI, −5.70 to 30.89%) relative to controls.

At baseline, caffeine intake was 91 (66) mg/d in the TRE participants, 90 (78) mg/d in the CR participants, and 89 (99) mg/d in the control participants. Caffeine intake did not differ over time or between groups. Diet quality and physical activity did not differ over time or between groups, as reported previously [9].

### 3.5. Sleep Measures

Changes in sleep measures are reported in Table 2 and Figure 1. At baseline, mean (SD) sleep quality was poor (PSQI scores > 5) in the TRE (7.6 ± 3.2), CR (8.2 ± 3.4), and control (9.4 ± 3.8) groups. The PSQI sleep quality score did not change significantly between groups by month 6.

At baseline, mean (SD) sleep duration was shorter than the recommended 7 h minimum per night [16] in the TRE (6:36 ± 1:00 h:min), CR (6:06 ± 2:00 h:min), and control (5:54 ± 1:36 h:min) groups. Sleep duration did not change significantly between groups by month 6. Wake time and sleep onset latency also did not change significantly between groups by the end of the study. As for bedtime, the TRE group was going to sleep later by month 6 (00:47 h:min; 95% CI, 00:03 to 01:32 h:min) relative to controls only.

At baseline, the insomnia severity score (SD) was 8.2 (5.2) for the TRE group and 9.3 (6.2) for the control group, indicating subclinical insomnia (ISI score 8–14), and 7.4 (5.5) in the CR group, indicating no clinical insomnia (ISI score 0–7). Insomnia severity scores did not change significantly between groups by month 6.

At baseline, the risk for obstructive sleep apnea was present in 65% of TRE participants, 40% of CR participants, and 79% of control participants. The risk of obstructive sleep apnea did not change significantly between groups by the end of the study. Changes in body weight and HbA1c were not related to any sleep measure in any group.

## 4. Discussion

To our knowledge, this is the first study to compare the effects of TRE to CR on sleep outcomes in adults with T2D. Our results show that TRE produced significant reductions in body weight and HbA1c levels after 6 months but no changes in sleep quality, duration, insomnia severity, or risk of obstructive sleep apnea versus CR and controls.

Sleep quality and duration (measured by PSQI) remained unchanged in all groups by the end of the study. This finding is similar to what has been reported in other TRE studies [17,18,19]. For instance, Parr et al. [19] reported no change in body weight or sleep quality or duration after one month of 9 h TRE (10 am–7 pm) in adults with obesity and T2D. Gabel et al. [18] showed that 3 months of 8 h TRE (10 am–6 pm) did not alter sleep duration or quality in adults with obesity (without diabetes) even with a 2.6% weight loss. Similarly, Cienfuegos et al. [17] reported no change in sleep duration or quality after a 2-month intervention of 4 h TRE (3–7 pm) and 6 h TRE (1–7 pm) versus controls in adults with obesity (without diabetes) with a 3.2% weight loss. It is possible that sleep duration and quality were not improved in any of these TRE trials because the degree of weight loss fell short of being clinically significant (>5% weight loss from baseline). In trials that reported greater weight loss, such as the DiRECT trial [20] (8.8% weight loss), sleep duration and quality increased over 24 months in adults with obesity and T2D. Evidence suggests that weight loss may improve sleep duration and quality by alleviating sleep-disordered breathing and reducing sleep fragmentation [7].

Insomnia severity was also assessed. At the beginning of the study, participants in the TRE and control groups portrayed sub-clinical insomnia, while participants in the CR group displayed no clinically significant insomnia. By month 6, no changes in insomnia severity were noted in either intervention group versus controls. This finding is not surprising, as our participants did not portray clinically significant insomnia at the onset of treatment; therefore, it would be unlikely for this sleep metric to improve. This lack of effect on insomnia severity has also been observed in other TRE trials that observed a similar degree of weight loss (~3%) [17,18].

Obstructive sleep apnea is highly prevalent in those with T2D [21]. In the present trial, 65–79% of participants in the TRE and control groups were at high risk of sleep apnea, while 40% of participants in the CR group were at high risk of this disorder. After 6 months, no significant changes in the risk of sleep apnea were noted. However, our interventions may not have resulted in sufficient weight loss to impact this sleep metric. Findings suggest that a minimum weight loss of 10% may be required to lower the risk of obstructive sleep apnea in individuals with obesity [22]. Indeed, the Sleep AHEAD study showed an improvement in obstructive sleep apnea (measured by apnea-hypopnea index) with a 10% weight loss after 1 year of intensive lifestyle intervention, in 264 participants with obesity and T2D [6].

Although we did not see improvements in sleep parameters, one suggested mechanism of how TRE could enhance sleep quality is through the improvement of circadian rhythm alignment. By restricting food consumption to daytime hours, intermittent fasting could reinforce the body’s peripheral circadian rhythms. This reinforcement may help restore the natural homeostasis of the internal clock, potentially leading to better sleep for individuals with irregular sleep patterns [23].

This study has several limitations. First, our sample size was small (n = 75), and our power calculation was based solely on changes in body weight. Thus, it is probable that our study lacked sufficient power to detect significant changes in sleep parameters. Second, self-report was used to assess all sleep outcomes. The study would have been improved by incorporating wrist actigraphy to obtain more objective measurements of rest and activity patterns. Third, participants were permitted to drink caffeinated beverages during their fasting window. Although total caffeine intake did not change significantly between groups by month 6, some participants may have consumed caffeine late into the evening, which may have impacted their sleep. Fourth, the study may have been too short (6 months) to produce any meaningful changes in body weight and sleep parameters.

In summary, the weight loss induced by 6 months of TRE does not improve sleep duration, quality, insomnia severity, or the risk of obstructive sleep apnea in those with obesity and T2D relative to CR and controls. However, a well-powered randomized controlled trial specifically designed to evaluate the effects of TRE on sleep in this population group will be needed to confirm these findings.

## Figures and Tables

**Table 1:** Baseline characteristics.

Variables	Time Restricted Eating (TRE)	Daily Calorie Restriction (CR)	Control (CON)
**n**	25	25	25
**Age (y)**	56 (13)	55 (13)	54 (11)
**Sex (no. female/male)**	18/7	17/8	18/7
**Diabetes duration (y)**	15 (11)	14 (9)	12 (9)
**Body weight & composition**			
Body weight (kg)	105 (25)	104 (17)	107 (22)
Fat mass (kg)	43 (10)	49 (10)	47 (14)
Lean mass (kg)	54 (13)	52 (10)	53 (10)
Visceral fat mass (kg)	1.9 (0.7)	2.5 (1.0)	2.2 (0.9)
Waist circumference (cm)	117 (13)	120 (9)	121 (15)
Body mass index (BMI; kg/m^2^)	39 (9)	38 (5)	39 (7)
**Glycemic control**			
HbA1c (%)	8.3 (2.0)	8.1 (1.5)	7.9 (1.3)
Time in euglycemic range (%)	63 (30)	60 (30)	62 (32)
**Pittsburgh Sleep Quality Index (PSQI)**			
Total PSQI sleep quality score	7.6 (3.2)	8.2 (3.4)	9.4 (3.8)
Wake time (h:min)	6:22 (1:31)	6:41 (1:35)	6:11 (2:14)
Bedtime (h:min)	23:46 (1:46)	00:35 (1:17)	00:17 (1:47)
Sleep duration (h:min)	6:36 (1:00)	6:06 (2:00)	5:54 (1:36)
Sleep onset latency score	1.3 (1.1)	1.3 (1.2)	1.9 (1.1)
**Insomnia severity index (ISI)**			
Total score	8.2 (5.2)	7.4 (5.5)	9.3 (6.2)
**Berlin questionnaire**			
High risk of obstructive sleep apnea (% occurrences)	65	40	79
**Morningness–Eveningness (MEQ)**			
Total score	58 (8)	55 (9)	54 (10)

Abbreviations: HbA1c: glycated hemoglobin, ISI: Insomnia Severity Index, PSQI: Pittsburgh Sleep Quality Index. Continuous variables are reported as mean (SD). Participants at a high risk of obstructive sleep apnea reported as % occurrences.

**Table 2:** Change in body weight, glycemia, and sleep parameters ^a^.

Variables	Change from Baseline to Month 6 (95% CI)			Difference between Groups by Month 6 (95% CI)		
	Time Restricted Eating (TRE)	Daily Calorie Restriction (CR)	Control (CON)	TRE vs. CR	TRE vs. CON	CR vs. CON
**Body weight and composition**						
Body weight (%)	**−4.28 (−6.26, −2.31) ^b^**	**−2.50 (−3.83, −1.18) ^b^**	**−0.72 (−2.14, 0.69)**	−1.78 (−4.09, 0.53)	**−3.56 (−5.92, −1.20) ^b^**	−1.78 (−3.67, 0.11)
Body weight (kg)	**−4.52 (−6.73, −2.30) ^b^**	**−2.63 (−3.99, −1.27) ^b^**	**−1.07 (−2.70, 0.57)**	−1.89 (−4.42, 0.64)	**−3.45 (−6.13, −0.77) ^b^**	−1.56 (−3.63, 0.51)
Fat mass (kg)	**−2.77 (−4.36, −1.18) ^c^**	**−1.92 (−3.20, −0.65) ^c^**	**−0.28 (−1.46, 0.90)**	−0.85 (−2.82, 1.13)	**−2.49 (−4.41, −0.58) ^c^**	−1.65 (−3.33, 0.04)
Lean mass (kg)	−0.97 (−2.04, 0.10)	−0.46 (−1.15, 0.23)	−0.59 (−1.49, 0.32)	−0.51 (−1.74, 0.72)	−0.38 (−1.74, 0.98)	0.13 (−0.98, 1.23)
Visceral fat mass (kg)	−0.16 (−0.36, 0.05)	**−0.18 (−0.32, −0.04) ^c^**	−0.07 (−0.18, 0.04)	0.02 (−0.21, 0.26)	−0.09 (−0.31, 0.13)	−0.11 (−0.28, 0.06)
Waist circumference (cm)	**−3.92 (−5.93, −1.91) ^c^**	**−3.97 (−6.03, −1.92) ^c^**	−0.48 (−1.66, 0.71)	0.05 (−2.74, 2.84)	**−3.44 (−5.71, −1.18) ^c^**	**−3.50 (−5.80, −1.20) ^c^**
**Glycemic control**						
HbA1c (%)	**−0.72 (−1.25, −0.18) ^c^**	**−0.75 (−1.20, −0.30) ^c^**	0.19 (−0.30, 0.68)	0.04 (−0.64, 0.72)	**−0.91 (−1.61, −0.20) ^c^**	**−0.94 (−1.59, −0.30) ^c^**
Time in euglycemic range (%)	4.78 (−3.52, 13.08)	**10.65 (2.65, 18.65)**	−7.81 (−24.90, 9.27)	−5.87 (−17.03, 5.28)	12.59 (−5.70, 30.89)	**18.46 (0.31, 36.62)**
**Pittsburgh Sleep Quality Index (PSQI)**						
Total PSQI sleep quality score	−0.60 (−1.84, 0.65)	−1.33 (−2.71, 0.05)	−0.65 (−2.15, 0.85)	0.73 (−1.07, 2.54)	0.06 (−1.84, 1.95)	−0.68 (−2.66, 1.30)
Wake time (h:min)	−00:05 (−00:35, 00:26)	00:04 (−00:28, 00:36)	00:17 (−00:24, 00:59)	−00:08 (−00:52, 00:34)	−00:22 (−01.12, 00:28)	−00:14 (−01:04, 00:37)
Bedtime (h:min)	00:21 (−00:12, 00:54)	00:14 (−00:27, 00:56)	−00:26 (−00:58, 00:05)	00:07 (−00:45, 00:58)	**00:47 (00:03, 01:32) ^c^**	00:41 (−00:10, 01:32)
Sleep duration (h:min)	−00:20 (−00:50, 00:09)	−00:05 (−00:47, 00:38)	00:14 (−00:31, 00:58)	−00:16 (−01:07, 00:35)	−00:34 (−01:25, 00:17)	−00:18 (−01:18, 00:42)
Sleep onset latency (h:min)	00:12 (−00:21, 00:44)	−00:12 (−00:42, 00:17)	−00:31 (−01:09, 00:06)	00:24 (−00:19, 01:07)	00:43 (−00:05, 01:31)	00:19 (−00:27, 01:05)
**Insomnia severity index (ISI)**						
Total score	−0.81 (−3.21, 1.60)	−0.31 (−2.02, 1.39)	−1.60 (−4.68, 1.48)	−0.49 (−3.34, 2.36)	0.80 (−3.00, 4.59)	1.29 (−2.13, 4.71)
**Berlin questionnaire**						
High risk of obstructive sleep apnea (% occurrences)	−13 (−28, 3)	−2 (−29, 25)	−34 (−56, −12)	−11 (−41, 20)	22 (−5, 48)	32 (−1, 66)

Abbreviations: HbA1c: glycated hemoglobin, ISI: Insomnia severity index, PSQI: Pittsburgh Sleep Quality Index. ^a^ Means were estimated using an intention-to-treat analysis using a linear mixed model with 95% CIs for each parameter from baseline by diet group. ^b^ Indicates statistical significance using Bonferroni-adjusted two-tailed *p* < 0.017. ^c^ Indicates statistical significance using *p* < 0.05.

## Data Availability

The original contributions presented in the study are included in the article, further inquiries can be directed to the corresponding authors.

## Trial Registration

ClinicalTrials.gov, NCT05225337.
